# {5,10,15,20-Tetra­kis[4-(hex­yloxy)phen­yl]porphyrinato}nickel(II)

**DOI:** 10.1107/S160053681202226X

**Published:** 2012-05-26

**Authors:** Yu-Fang Wang, Hong-Bin Zhao, Liang Chen, De-Liang Yang, Bang-Ying Wang

**Affiliations:** a Department of Organic Chemistry, the College of Chemistry, Xiangtan University, Hunan 411105, People’s Republic of China; b College of Chemistry and Environmental Engineering, Dongguan University of Technology, Guangdong 523808, People’s Republic of China

## Abstract

The mol­ecule of the title compound, [Ni(C_68_H_76_N_4_O_4_)], is located on a crystallographic inversion center. The Ni—N distances within the square-shaped coordination environment are 1.951 (2) and 1.954 (2) Å. Three terminal C atoms in one of the hexyl groups are disordered over two sets of sites, with site-occupancy factors of 0.615 (13) and 0.385 (13).

## Related literature

For related structures, see: Scheidt (1977 ▶); Maclean *et al.* (1996 ▶); Jentzen *et al.* (1996 ▶); Chen *et al.* (2010 ▶). For potential applications of porphyrins and metalloporphyrins in liquid crystals, prototypical multistate counters and artificial light-harvesting antennas, see: Castella *et al.* (2002 ▶); Schweikart *et al.* (2002 ▶); Imahori (2004 ▶). For their applications in dye-sensitised solar cells, see: Barea *et al.* (2011 ▶); Yella *et al.* (2011 ▶) and for their applications in enzyme mimics, see: Anderson & Sanders (1995 ▶).
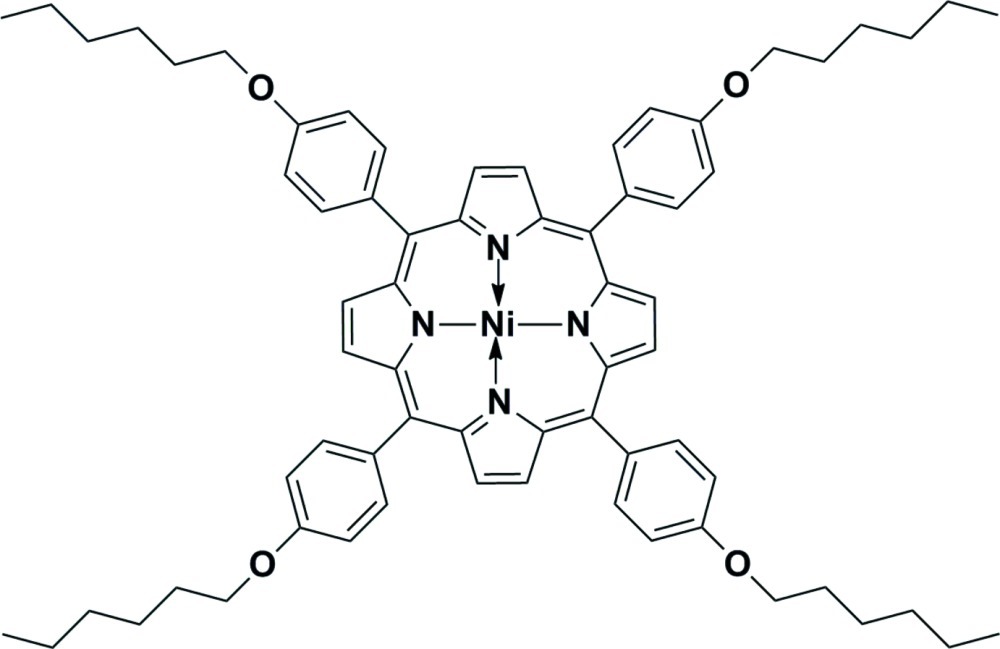


## Experimental

### 

#### Crystal data

[Ni(C_68_H_76_N_4_O_4_)]
*M* *_r_* = 1072.04
Orthorhombic, 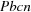

*a* = 18.7735 (10) Å
*b* = 10.8439 (6) Å
*c* = 28.7173 (16) Å
*V* = 5846.2 (6) Å^3^
*Z* = 4
Mo *K*α radiation
μ = 0.38 mm^−1^
*T* = 185 K
0.27 × 0.19 × 0.10 mm

#### Data collection

Bruker APEX CCD diffractometer
Absorption correction: multi-scan (*SADABS*; Sheldrick, 2004 ▶) *T*_min_ = 0.904, *T*_max_ = 0.963
30888 measured reflections
5173 independent reflections
3950 reflections with *I* > 2σ(*I*)
*R*_int_ = 0.031

#### Refinement

*R*[*F* ^2^ > 2σ(*F* ^2^)] = 0.056
*wR*(*F* ^2^) = 0.156
*S* = 1.03
5173 reflections
380 parameters
10 restraints
H-atom parameters constrained
Δρ_max_ = 0.92 e Å^−3^
Δρ_min_ = −0.32 e Å^−3^

### 

Data collection: *SMART* (Bruker, 2002 ▶); cell refinement: *SAINT* (Bruker, 2002 ▶); data reduction: *SAINT*; program(s) used to solve structure: *SHELXS97* (Sheldrick, 2008 ▶); program(s) used to refine structure: *SHELXL97* (Sheldrick, 2008 ▶); molecular graphics: *SHELXTL* (Sheldrick, 2008 ▶); software used to prepare material for publication: *SHELXTL*.

## Supplementary Material

Crystal structure: contains datablock(s) global, I. DOI: 10.1107/S160053681202226X/bt5904sup1.cif

Structure factors: contains datablock(s) I. DOI: 10.1107/S160053681202226X/bt5904Isup2.hkl

Additional supplementary materials:  crystallographic information; 3D view; checkCIF report

## Figures and Tables

**Fig. 1.**
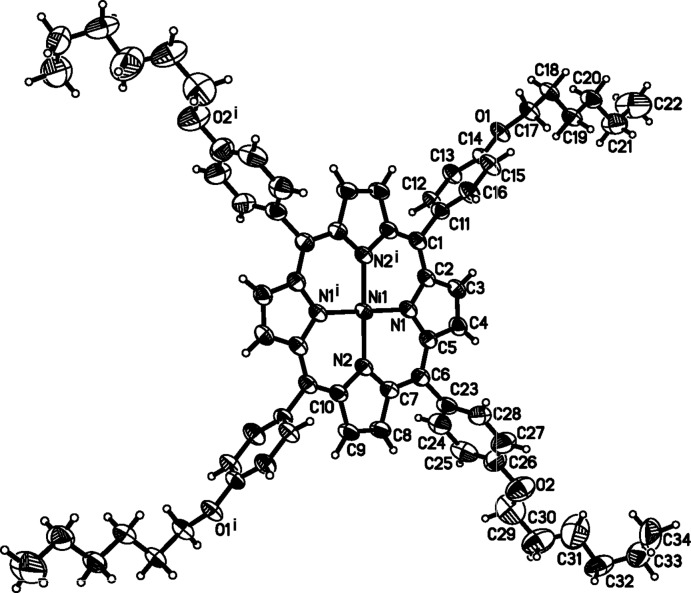
The molecular structure of the title compound with the atom numbering scheme. Displacement ellipsoids are drawn at the 50% probability level. The minor occupied atoms of the disordered group are not shown. Symmetry code: (i) -x, 1 - y, -z.

## supplementary crystallographic information

### Comment

Porphyrins and metalloporphyrins are researched in many aspects, e.g. as liquid crystals (Castella *et al.*, 2002), prototypical multistate counters (Schweikart *et al.*, 2002), artificial light-harvesting antennas (Imahori,2004), dye-sensitized solar cells (Barea *et al.*,2011; Yella *et al.*, 2011) or enzyme mimics (Anderson *et al.*, 1995).

The porphyrin moiety is essentially planar, the macrocyclic core is planar with a mean deviation of 0.0244 Å. The four-coordinate Ni ion is located on the inversion center with Ni–N distances of 1.950 (3) and 1.954 (2) Å. These bond lengths are in agreement with those found in other nickel porphyrin compounds (Scheidt, 1977; Maclean *et al.*, 1996; Jentzen *et al.*, 1996; Chen *et al.*, 2010.).

Three terminal C atoms (C32, C33 & C34) in one of the hexyl group are disordered over two positions with site occupancy factors of 0.615 (13) and 0.385 (13).

### Experimental

0.04 mmol *meso*-tetrakis[*p*-(hexyloxy)phenyl] porphyrin and 0.40 mmol Ni(CH_3_COO_)2_.4H_2_O were dissolved in 20 ml chloroform, refluxed for 8 h, and the solvent was removed by a rotary evaporator, the residue was purified by column chromatography with chloroform, then crystallized by methanol and chloroform, and a purple solid was obtained (yield = 68%). Single crystals were recrystallization from a dichloromethane solution at room temperature.

### Refinement

H atoms were positioned geometrically and refined using a riding model, with C—H = 0.95 (aromatic), 0.99 (CH_2_) and 0.98 (CH_3_) Å and with *U*_iso_(H) = 1.2(1.5 for methyl)*U*_eq_(C). Three terminal C atoms (C32, C33 & C34) in the hexyl group are disordered over two positions. The occupancy factors were refined to a ratio of 0.615 (13):0.385 (13). The C—C bonds were restrained to 1.52 (1)Å and 1-3 distances to 2.48 (1) Å.

### Crystal data

**Table:**

[Ni(C_68_H_76_N_4_O_4_)]	*D*_x_ = 1.218 Mg m^−^^3^
*M**_r_* = 1072.04	Mo *K*α radiation, λ = 0.71073 Å
Orthorhombic, *P**b**c**n*	Cell parameters from 7739 reflections
*a* = 18.7735 (10) Å	θ = 2.3–25.0°
*b* = 10.8439 (6) Å	µ = 0.38 mm^−^^1^
*c* = 28.7173 (16) Å	*T* = 185 K
*V* = 5846.2 (6) Å^3^	Block, purple
*Z* = 4	0.27 × 0.19 × 0.10 mm
*F*(000) = 2288	

### Data collection

**Table:**

Bruker APEX CCD diffractometer	5173 independent reflections
Radiation source: fine-focus sealed tube	3950 reflections with *I* > 2σ(*I*)
Graphite monochromator	*R*_int_ = 0.031
φ and ω scans	θ_max_ = 25.0°, θ_min_ = 1.8°
Absorption correction: multi-scan (*SADABS*; Sheldrick, 2004)	*h* = −22→11
*T*_min_ = 0.904, *T*_max_ = 0.963	*k* = −12→12
30888 measured reflections	*l* = −34→34

### Refinement

**Table:**

Refinement on *F*^2^	Primary atom site location: structure-invariant direct methods
Least-squares matrix: full	Secondary atom site location: difference Fourier map
*R*[*F*^2^ > 2σ(*F*^2^)] = 0.056	Hydrogen site location: inferred from neighbouring sites
*wR*(*F*^2^) = 0.156	H-atom parameters constrained
*S* = 1.03	*w* = 1/[σ^2^(*F*_o_^2^) + (0.0695*P*)^2^ + 7.6994*P*] where *P* = (*F*_o_^2^ + 2*F*_c_^2^)/3
5173 reflections	(Δ/σ)_max_ = 0.005
380 parameters	Δρ_max_ = 0.92 e Å^−^^3^
10 restraints	Δρ_min_ = −0.32 e Å^−^^3^

### Special details

**Table:**

Geometry. All e.s.d.'s (except the e.s.d. in the dihedral angle between two l.s. planes) are estimated using the full covariance matrix. The cell e.s.d.'s are taken into account individually in the estimation of e.s.d.'s in distances, angles and torsion angles; correlations between e.s.d.'s in cell parameters are only used when they are defined by crystal symmetry. An approximate (isotropic) treatment of cell e.s.d.'s is used for estimating e.s.d.'s involving l.s. planes.
Refinement. Refinement of *F*^2^ against ALL reflections. The weighted *R*-factor *wR* and goodness of fit *S* are based on *F*^2^, conventional *R*-factors *R* are based on *F*, with *F* set to zero for negative *F*^2^. The threshold expression of *F*^2^ > σ(*F*^2^) is used only for calculating *R*-factors(gt) *etc*. and is not relevant to the choice of reflections for refinement. *R*-factors based on *F*^2^ are statistically about twice as large as those based on *F*, and *R*- factors based on ALL data will be even larger.

### Fractional atomic coordinates and isotropic or equivalent isotropic displacement parameters (Å^2^)

**Table:**

	*x*	*y*	*z*	*U*_iso_*/*U*_eq_	Occ. (<1)
Ni1	0.0000	0.5000	0.0000	0.03368 (17)	
N1	0.05923 (12)	0.4241 (2)	0.04806 (8)	0.0365 (6)	
N2	−0.08523 (12)	0.4574 (2)	0.03539 (8)	0.0360 (5)	
O1	0.47501 (10)	0.4148 (2)	0.05027 (8)	0.0470 (6)	
O2	−0.1075 (2)	0.0150 (3)	0.24746 (10)	0.0912 (11)	
C1	0.17914 (15)	0.4636 (3)	0.01680 (11)	0.0383 (7)	
C2	0.13284 (15)	0.4180 (3)	0.04994 (10)	0.0389 (7)	
C3	0.15575 (16)	0.3543 (3)	0.09105 (10)	0.0433 (7)	
H3	0.2036	0.3402	0.1004	0.052*	
C4	0.09688 (16)	0.3184 (3)	0.11395 (11)	0.0449 (8)	
H4	0.0952	0.2728	0.1422	0.054*	
C5	0.03718 (15)	0.3619 (3)	0.08767 (10)	0.0389 (7)	
C6	−0.03289 (16)	0.3440 (3)	0.10139 (10)	0.0402 (7)	
C7	−0.08952 (15)	0.3932 (3)	0.07679 (10)	0.0400 (7)	
C8	−0.16234 (16)	0.3787 (3)	0.09132 (11)	0.0466 (8)	
H8	−0.1787	0.3383	0.1186	0.056*	
C9	−0.20280 (16)	0.4336 (3)	0.05864 (11)	0.0458 (8)	
H9	−0.2533	0.4401	0.0588	0.055*	
C10	−0.15578 (15)	0.4803 (3)	0.02365 (11)	0.0375 (7)	
C11	0.25789 (15)	0.4496 (3)	0.02455 (10)	0.0395 (7)	
C12	0.29479 (16)	0.5322 (3)	0.05231 (11)	0.0442 (8)	
H12	0.2700	0.5987	0.0665	0.053*	
C13	0.36741 (16)	0.5192 (3)	0.05977 (11)	0.0442 (8)	
H13	0.3920	0.5768	0.0788	0.053*	
C14	0.40400 (15)	0.4222 (3)	0.03951 (10)	0.0397 (7)	
C15	0.36835 (16)	0.3412 (3)	0.01064 (12)	0.0498 (8)	
H15	0.3934	0.2763	−0.0044	0.060*	
C16	0.29529 (17)	0.3554 (3)	0.00380 (12)	0.0502 (8)	
H16	0.2708	0.2987	−0.0157	0.060*	
C17	0.51606 (16)	0.3211 (3)	0.02732 (12)	0.0483 (8)	
H17A	0.5212	0.3410	−0.0062	0.058*	
H17B	0.4919	0.2402	0.0301	0.058*	
C18	0.58842 (15)	0.3160 (3)	0.05030 (11)	0.0478 (8)	
H18A	0.6074	0.4008	0.0532	0.057*	
H18B	0.6214	0.2685	0.0303	0.057*	
C19	0.58589 (16)	0.2569 (3)	0.09827 (12)	0.0506 (8)	
H19A	0.5524	0.3041	0.1180	0.061*	
H19B	0.5669	0.1721	0.0952	0.061*	
C20	0.65764 (17)	0.2511 (4)	0.12263 (12)	0.0565 (9)	
H20A	0.6921	0.2087	0.1020	0.068*	
H20B	0.6751	0.3362	0.1277	0.068*	
C21	0.6552 (2)	0.1846 (5)	0.16901 (15)	0.0810 (13)	
H21A	0.6363	0.1004	0.1641	0.097*	
H21B	0.6220	0.2286	0.1900	0.097*	
C22	0.7278 (3)	0.1760 (7)	0.1922 (2)	0.143 (3)	
H22A	0.7617	0.1372	0.1708	0.215*	
H22B	0.7240	0.1263	0.2206	0.215*	
H22C	0.7445	0.2590	0.2002	0.215*	
C23	−0.04850 (16)	0.2638 (3)	0.14258 (11)	0.0470 (8)	
C24	−0.06468 (19)	0.3105 (4)	0.18589 (12)	0.0599 (10)	
H24	−0.0634	0.3970	0.1911	0.072*	
C25	−0.08340 (19)	0.2286 (5)	0.22295 (12)	0.0676 (12)	
H25	−0.0939	0.2595	0.2531	0.081*	
C26	−0.0859 (2)	0.1039 (4)	0.21393 (15)	0.0678 (11)	
C27	−0.0687 (2)	0.0577 (4)	0.17204 (15)	0.0728 (11)	
H27	−0.0694	−0.0289	0.1670	0.087*	
C28	−0.05038 (19)	0.1361 (4)	0.13678 (14)	0.0616 (10)	
H28	−0.0385	0.1024	0.1072	0.074*	
C29	−0.1321 (3)	0.0598 (5)	0.28906 (16)	0.1011 (17)	
H29A	−0.1692	0.1231	0.2838	0.121*	
H29B	−0.0928	0.0972	0.3071	0.121*	
C30	−0.1638 (3)	−0.0529 (6)	0.31558 (17)	0.1079 (19)	
H30A	−0.1854	−0.0229	0.3449	0.129*	
H30B	−0.2026	−0.0884	0.2964	0.129*	
C31	−0.1156 (4)	−0.1477 (5)	0.3265 (2)	0.143 (3)	
H31A	−0.0793	−0.1165	0.3486	0.172*	0.615 (13)
H31B	−0.0909	−0.1752	0.2979	0.172*	0.615 (13)
H31C	−0.0758	−0.1019	0.3411	0.172*	0.385 (13)
H31D	−0.0975	−0.1720	0.2954	0.172*	0.385 (13)
C32A	−0.1552 (5)	−0.2553 (7)	0.3481 (3)	0.090 (3)	0.615 (13)
H32C	−0.1738	−0.2315	0.3791	0.109*	0.615 (13)
H32D	−0.1960	−0.2788	0.3282	0.109*	0.615 (13)
C33A	−0.1051 (5)	−0.3617 (8)	0.3529 (4)	0.105 (4)	0.615 (13)
H33A	−0.0625	−0.3350	0.3705	0.126*	0.615 (13)
H33B	−0.1286	−0.4283	0.3708	0.126*	0.615 (13)
C34A	−0.0823 (12)	−0.4111 (15)	0.3060 (6)	0.189 (12)	0.615 (13)
H34A	−0.1233	−0.4489	0.2904	0.284*	0.615 (13)
H34B	−0.0449	−0.4731	0.3103	0.284*	0.615 (13)
H34C	−0.0640	−0.3434	0.2869	0.284*	0.615 (13)
C32B	−0.1148 (13)	−0.2696 (12)	0.3530 (7)	0.201 (16)	0.385 (13)
H32E	−0.0653	−0.3000	0.3557	0.241*	0.385 (13)
H32F	−0.1337	−0.2567	0.3848	0.241*	0.385 (13)
C33B	−0.1599 (8)	−0.3646 (10)	0.3279 (6)	0.111 (7)	0.385 (13)
H33C	−0.1948	−0.3227	0.3075	0.133*	0.385 (13)
H33D	−0.1864	−0.4150	0.3508	0.133*	0.385 (13)
C34B	−0.1117 (18)	−0.447 (2)	0.2988 (8)	0.156 (14)	0.385 (13)
H34D	−0.0900	−0.3987	0.2737	0.233*	0.385 (13)
H34E	−0.1398	−0.5144	0.2853	0.233*	0.385 (13)
H34F	−0.0741	−0.4818	0.3186	0.233*	0.385 (13)

### Atomic displacement parameters (Å^2^)

**Table:**

	*U*^11^	*U*^22^	*U*^33^	*U*^12^	*U*^13^	*U*^23^
Ni1	0.0224 (3)	0.0412 (3)	0.0374 (3)	0.0014 (2)	−0.0036 (2)	−0.0020 (2)
N1	0.0252 (12)	0.0438 (14)	0.0405 (13)	0.0027 (10)	−0.0047 (10)	−0.0028 (11)
N2	0.0257 (12)	0.0418 (13)	0.0406 (13)	0.0017 (10)	−0.0042 (10)	−0.0026 (11)
O1	0.0249 (10)	0.0562 (14)	0.0599 (13)	0.0074 (10)	−0.0073 (10)	−0.0135 (11)
O2	0.114 (3)	0.089 (2)	0.0700 (19)	−0.023 (2)	0.0205 (17)	−0.0054 (17)
C1	0.0277 (15)	0.0418 (16)	0.0455 (16)	0.0025 (12)	−0.0053 (13)	−0.0065 (14)
C2	0.0281 (15)	0.0448 (17)	0.0439 (16)	0.0041 (13)	−0.0045 (13)	−0.0051 (14)
C3	0.0299 (16)	0.0527 (19)	0.0473 (17)	0.0071 (14)	−0.0075 (13)	−0.0021 (15)
C4	0.0359 (17)	0.0529 (19)	0.0459 (17)	0.0058 (14)	−0.0061 (14)	0.0045 (15)
C5	0.0333 (16)	0.0427 (17)	0.0408 (16)	0.0022 (13)	−0.0051 (13)	−0.0021 (13)
C6	0.0340 (16)	0.0445 (17)	0.0422 (16)	0.0006 (13)	−0.0028 (13)	−0.0018 (14)
C7	0.0309 (15)	0.0451 (17)	0.0439 (16)	−0.0003 (13)	−0.0004 (13)	−0.0009 (14)
C8	0.0309 (16)	0.062 (2)	0.0471 (18)	−0.0005 (15)	0.0022 (14)	0.0050 (16)
C9	0.0257 (15)	0.060 (2)	0.0517 (18)	−0.0008 (14)	−0.0001 (14)	0.0008 (16)
C10	0.0280 (14)	0.0392 (16)	0.0453 (17)	0.0004 (12)	−0.0016 (13)	−0.0038 (13)
C11	0.0274 (15)	0.0486 (17)	0.0424 (16)	0.0019 (13)	−0.0026 (13)	0.0016 (14)
C12	0.0286 (15)	0.0517 (19)	0.0524 (18)	0.0080 (13)	−0.0030 (14)	−0.0082 (15)
C13	0.0321 (16)	0.0498 (19)	0.0508 (18)	0.0014 (13)	−0.0082 (14)	−0.0121 (15)
C14	0.0243 (14)	0.0501 (18)	0.0447 (16)	0.0041 (13)	−0.0037 (12)	−0.0008 (14)
C15	0.0319 (17)	0.057 (2)	0.061 (2)	0.0085 (15)	−0.0066 (14)	−0.0185 (17)
C16	0.0334 (17)	0.056 (2)	0.061 (2)	0.0039 (15)	−0.0116 (15)	−0.0188 (17)
C17	0.0322 (17)	0.059 (2)	0.0535 (19)	0.0081 (15)	−0.0008 (14)	−0.0086 (16)
C18	0.0274 (15)	0.061 (2)	0.0554 (19)	0.0092 (14)	−0.0006 (14)	−0.0037 (16)
C19	0.0324 (17)	0.060 (2)	0.059 (2)	0.0016 (15)	0.0011 (15)	−0.0034 (17)
C20	0.0341 (18)	0.078 (3)	0.058 (2)	0.0042 (17)	−0.0023 (15)	0.0047 (18)
C21	0.053 (2)	0.120 (4)	0.070 (3)	−0.001 (2)	−0.007 (2)	0.027 (3)
C22	0.078 (4)	0.257 (9)	0.094 (4)	0.002 (5)	−0.025 (3)	0.072 (5)
C23	0.0287 (16)	0.068 (2)	0.0445 (17)	0.0011 (15)	−0.0028 (13)	0.0063 (16)
C24	0.050 (2)	0.081 (3)	0.048 (2)	0.0041 (19)	−0.0055 (16)	0.0026 (19)
C25	0.046 (2)	0.119 (4)	0.0381 (18)	0.004 (2)	−0.0017 (16)	−0.006 (2)
C26	0.063 (3)	0.068 (3)	0.073 (3)	−0.008 (2)	−0.003 (2)	0.013 (2)
C27	0.077 (3)	0.073 (3)	0.068 (3)	−0.010 (2)	0.017 (2)	0.006 (2)
C28	0.052 (2)	0.069 (3)	0.065 (2)	−0.0036 (19)	0.0091 (18)	0.011 (2)
C29	0.114 (4)	0.127 (5)	0.062 (3)	0.010 (4)	−0.013 (3)	−0.023 (3)
C30	0.136 (5)	0.125 (5)	0.062 (3)	−0.029 (4)	0.026 (3)	0.007 (3)
C31	0.220 (8)	0.111 (5)	0.098 (4)	0.031 (6)	0.063 (5)	0.008 (4)
C32A	0.107 (8)	0.085 (6)	0.079 (6)	−0.030 (6)	0.038 (5)	−0.005 (5)
C33A	0.090 (7)	0.099 (7)	0.125 (8)	0.003 (6)	−0.001 (6)	0.052 (7)
C34A	0.22 (2)	0.085 (10)	0.27 (2)	0.070 (14)	0.137 (18)	0.050 (11)
C32B	0.19 (3)	0.14 (2)	0.27 (3)	−0.03 (2)	−0.17 (2)	0.04 (2)
C33B	0.113 (13)	0.107 (14)	0.112 (12)	−0.014 (10)	−0.041 (10)	0.044 (11)
C34B	0.27 (4)	0.108 (18)	0.094 (13)	−0.04 (2)	0.039 (18)	0.011 (11)

### Geometric parameters (Å, º)

**Table:**

Ni1—N2^i^	1.951 (2)	C20—H20A	0.9900
Ni1—N2	1.951 (2)	C20—H20B	0.9900
Ni1—N1^i^	1.954 (2)	C21—C22	1.520 (6)
Ni1—N1	1.954 (2)	C21—H21A	0.9900
N1—C2	1.385 (4)	C21—H21B	0.9900
N1—C5	1.386 (4)	C22—H22A	0.9800
N2—C7	1.380 (4)	C22—H22B	0.9800
N2—C10	1.389 (4)	C22—H22C	0.9800
O1—C14	1.371 (3)	C23—C24	1.377 (5)
O1—C17	1.436 (4)	C23—C28	1.396 (5)
O2—C29	1.370 (5)	C24—C25	1.431 (5)
O2—C26	1.421 (5)	C24—H24	0.9500
C1—C2	1.381 (4)	C25—C26	1.378 (6)
C1—C10^i^	1.383 (4)	C25—H25	0.9500
C1—C11	1.503 (4)	C26—C27	1.342 (6)
C2—C3	1.434 (4)	C27—C28	1.367 (5)
C3—C4	1.344 (4)	C27—H27	0.9500
C3—H3	0.9500	C28—H28	0.9500
C4—C5	1.431 (4)	C29—C30	1.558 (7)
C4—H4	0.9500	C29—H29A	0.9900
C5—C6	1.387 (4)	C29—H29B	0.9900
C6—C7	1.383 (4)	C30—C31	1.405 (8)
C6—C23	1.497 (4)	C30—H30A	0.9900
C7—C8	1.438 (4)	C30—H30B	0.9900
C8—C9	1.346 (4)	C31—C32A	1.517 (7)
C8—H8	0.9500	C31—C32B	1.526 (10)
C9—C10	1.430 (4)	C31—H31A	0.9900
C9—H9	0.9500	C31—H31B	0.9900
C10—C1^i^	1.383 (4)	C31—H31C	0.9900
C11—C16	1.375 (4)	C31—H31D	0.9900
C11—C12	1.385 (4)	C32A—C33A	1.496 (8)
C12—C13	1.387 (4)	C32A—H32C	0.9900
C12—H12	0.9500	C32A—H32D	0.9900
C13—C14	1.384 (4)	C33A—C34A	1.510 (9)
C13—H13	0.9500	C33A—H33A	0.9900
C14—C15	1.381 (4)	C33A—H33B	0.9900
C15—C16	1.394 (4)	C34A—H34A	0.9800
C15—H15	0.9500	C34A—H34B	0.9800
C16—H16	0.9500	C34A—H34C	0.9800
C17—C18	1.511 (4)	C32B—C33B	1.516 (9)
C17—H17A	0.9900	C32B—H32E	0.9900
C17—H17B	0.9900	C32B—H32F	0.9900
C18—C19	1.520 (5)	C33B—C34B	1.524 (10)
C18—H18A	0.9900	C33B—H33C	0.9900
C18—H18B	0.9900	C33B—H33D	0.9900
C19—C20	1.519 (4)	C34B—H34D	0.9800
C19—H19A	0.9900	C34B—H34E	0.9800
C19—H19B	0.9900	C34B—H34F	0.9800
C20—C21	1.515 (5)		
			
N2^i^—Ni1—N2	180.0	C20—C21—C22	112.8 (4)
N2^i^—Ni1—N1^i^	89.94 (10)	C20—C21—H21A	109.0
N2—Ni1—N1^i^	90.06 (10)	C22—C21—H21A	109.0
N2^i^—Ni1—N1	90.06 (10)	C20—C21—H21B	109.0
N2—Ni1—N1	89.94 (10)	C22—C21—H21B	109.0
N1^i^—Ni1—N1	180.0	H21A—C21—H21B	107.8
C2—N1—C5	104.0 (2)	C21—C22—H22A	109.5
C2—N1—Ni1	128.0 (2)	C21—C22—H22B	109.5
C5—N1—Ni1	127.94 (19)	H22A—C22—H22B	109.5
C7—N2—C10	104.1 (2)	C21—C22—H22C	109.5
C7—N2—Ni1	128.03 (19)	H22A—C22—H22C	109.5
C10—N2—Ni1	127.8 (2)	H22B—C22—H22C	109.5
C14—O1—C17	117.4 (2)	C24—C23—C28	117.9 (3)
C29—O2—C26	116.5 (4)	C24—C23—C6	122.9 (3)
C2—C1—C10^i^	122.5 (3)	C28—C23—C6	119.1 (3)
C2—C1—C11	118.8 (3)	C23—C24—C25	119.9 (4)
C10^i^—C1—C11	118.7 (3)	C23—C24—H24	120.1
C1—C2—N1	125.7 (3)	C25—C24—H24	120.1
C1—C2—C3	123.5 (3)	C26—C25—C24	118.6 (4)
N1—C2—C3	110.8 (3)	C26—C25—H25	120.7
C4—C3—C2	107.2 (3)	C24—C25—H25	120.7
C4—C3—H3	126.4	C27—C26—C25	121.8 (4)
C2—C3—H3	126.4	C27—C26—O2	115.0 (4)
C3—C4—C5	106.9 (3)	C25—C26—O2	123.2 (4)
C3—C4—H4	126.6	C26—C27—C28	119.5 (4)
C5—C4—H4	126.6	C26—C27—H27	120.3
N1—C5—C6	125.8 (3)	C28—C27—H27	120.3
N1—C5—C4	111.1 (3)	C27—C28—C23	122.4 (4)
C6—C5—C4	123.2 (3)	C27—C28—H28	118.8
C7—C6—C5	122.0 (3)	C23—C28—H28	118.8
C7—C6—C23	118.5 (3)	O2—C29—C30	106.1 (4)
C5—C6—C23	119.4 (3)	O2—C29—H29A	110.5
N2—C7—C6	126.1 (3)	C30—C29—H29A	110.5
N2—C7—C8	111.1 (3)	O2—C29—H29B	110.5
C6—C7—C8	122.7 (3)	C30—C29—H29B	110.5
C9—C8—C7	106.6 (3)	H29A—C29—H29B	108.7
C9—C8—H8	126.7	C31—C30—C29	115.8 (5)
C7—C8—H8	126.7	C31—C30—H30A	108.3
C8—C9—C10	107.3 (3)	C29—C30—H30A	108.3
C8—C9—H9	126.3	C31—C30—H30B	108.3
C10—C9—H9	126.3	C29—C30—H30B	108.3
C1^i^—C10—N2	125.8 (3)	H30A—C30—H30B	107.4
C1^i^—C10—C9	123.4 (3)	C30—C31—C32A	109.7 (7)
N2—C10—C9	110.8 (3)	C30—C31—C32B	138.6 (12)
C16—C11—C12	118.3 (3)	C30—C31—H31A	109.7
C16—C11—C1	120.9 (3)	C32A—C31—H31A	109.7
C12—C11—C1	120.8 (3)	C30—C31—H31B	109.7
C11—C12—C13	121.0 (3)	C32A—C31—H31B	109.7
C11—C12—H12	119.5	H31A—C31—H31B	108.2
C13—C12—H12	119.5	C30—C31—H31C	102.4
C14—C13—C12	120.0 (3)	C32B—C31—H31C	102.4
C14—C13—H13	120.0	C30—C31—H31D	102.4
C12—C13—H13	120.0	C32A—C31—H31D	109.4
O1—C14—C15	124.7 (3)	C32B—C31—H31D	102.4
O1—C14—C13	115.6 (3)	H31C—C31—H31D	104.9
C15—C14—C13	119.7 (3)	C33A—C32A—C31	108.8 (6)
C14—C15—C16	119.4 (3)	C33A—C32A—H32C	109.9
C14—C15—H15	120.3	C31—C32A—H32C	109.9
C16—C15—H15	120.3	C33A—C32A—H32D	109.9
C11—C16—C15	121.6 (3)	C31—C32A—H32D	109.9
C11—C16—H16	119.2	H32C—C32A—H32D	108.3
C15—C16—H16	119.2	C34A—C33A—C32A	111.8 (7)
O1—C17—C18	107.9 (3)	C34A—C33A—H33A	109.3
O1—C17—H17A	110.1	C32A—C33A—H33A	109.3
C18—C17—H17A	110.1	C34A—C33A—H33B	109.3
O1—C17—H17B	110.1	C32A—C33A—H33B	109.3
C18—C17—H17B	110.1	H33A—C33A—H33B	107.9
H17A—C17—H17B	108.4	C33B—C32B—C31	110.3 (8)
C17—C18—C19	112.5 (3)	C33B—C32B—H32E	109.6
C17—C18—H18A	109.1	C31—C32B—H32E	109.6
C19—C18—H18A	109.1	C33B—C32B—H32F	109.6
C17—C18—H18B	109.1	C31—C32B—H32F	109.6
C19—C18—H18B	109.1	H32E—C32B—H32F	108.1
H18A—C18—H18B	107.8	C32B—C33B—C34B	109.2 (9)
C20—C19—C18	114.0 (3)	C32B—C33B—H33C	109.8
C20—C19—H19A	108.7	C34B—C33B—H33C	109.8
C18—C19—H19A	108.7	C32B—C33B—H33D	109.8
C20—C19—H19B	108.7	C34B—C33B—H33D	109.8
C18—C19—H19B	108.7	H33C—C33B—H33D	108.3
H19A—C19—H19B	107.6	C33B—C34B—H34D	109.5
C21—C20—C19	113.4 (3)	C33B—C34B—H34E	109.5
C21—C20—H20A	108.9	H34D—C34B—H34E	109.5
C19—C20—H20A	108.9	C33B—C34B—H34F	109.5
C21—C20—H20B	108.9	H34D—C34B—H34F	109.5
C19—C20—H20B	108.9	H34E—C34B—H34F	109.5
H20A—C20—H20B	107.7		

Symmetry code: (i) −*x*, −*y*+1, −*z*.
